# Perceived Need and Social Relatedness Contribute to Change in Selective Prevention for Mental Illness: a Mixed Methods Study

**DOI:** 10.1007/s11121-025-01831-w

**Published:** 2025-08-12

**Authors:** Anne Dorothee Müller, Ida C. T. Gjøde, Sofie H. Christensen, Sophie K. Jørgensen, Kirstine Fischer, Mala Moszkowicz, Nicoline Hemager, Merete Nordentoft, Geneviève Piché, Anne A. E. Thorup

**Affiliations:** 1 Child and Adolescent Mental Health Center, Copenhagen University Hospital – Mental Health Services CPH, Copenhagen, Denmark; 2 Department of Clinical Medicine, University of Copenhagen, Copenhagen, Denmark; 3 Copenhagen Research Centre for Mental Health (CORE), Mental Health Services in the Capital Region of Denmark, Copenhagen, Denmark; 4 Department of Psychology, University of Copenhagen, Copenhagen, Denmark; 5 Centre de Recherche Universitaire Sur Les Jeunes Et Les Familles (CRUJeF), Québec, Canada; 6 Department of Psychoeducation and Psychology, Université du Québec en Outaouais, Saint-Jérôme, PQ Canada

**Keywords:** Child of impaired parents, Preventive psychiatry, Mechanisms of change, Familial high-risk children, Family-based intervention

## Abstract

Selective preventive interventions aim to reduce mental illness in high-risk populations, yet the reasons why some children benefit while others do not remain unclear. This study explores participants’ perceptions of mechanisms contributing to change in a family-based preventive intervention for children of parents with severe mental illness. Using an exploratory sequential mixed methods approach, we conducted an abductive qualitative analysis of focus groups (eight parents, eight children) to identify narratives of mechanisms contributing to change. The qualitative findings informed a subgrouping variable for a quantitative post hoc exploratory subgroup analysis of secondary data from the VIA Family trial (*N* = 110). The qualitative findings indicate that child mental health problems, parents’ personal unmet needs from childhood, children’s relatedness to peers and family, and contextual family-focused activities contribute to change within selective prevention. Quantitative results indicated that parents motivated by a need for support at baseline exhibited meaningful improvements in the home environment upon enrollment in the experimental preventive intervention compared with families motivated to support science (mean change: 5.07, 95% CI 2.11 to 8.03). However, no significant subgroup differences were observed in changes in children’s global functioning between the allocation groups. Parents’ perceived need for support facilitated engagement and home improvements, while children’s relatedness to peers and family contributed to their intervention experiences. These findings emphasize the importance of motivation and social connections in intervention outcomes, contributing to the growing field of precision prevention. Future research should explore these mechanisms as potential mediators or mechanisms of action for selective prevention. ClinicalTrial.gov Identifier: NCT03497663.

**Supplementary Information:**

The online version contains supplementary material available at 10.1007/s11121-025-01831-w.

## Introduction

Prevention of mental illness is not one-size-fits-all. Individuals differ in their risk profiles, needs, and responses to preventive interventions (World Health Organization, 2022). Precision prevention is an emerging approach in mental health sciences that seeks to tailor interventions by identifying what works best, for whom, and under what conditions (Ridenour, 2019). This personalized strategy can enhance various prevention efforts, including selective prevention, which targets individuals at higher risk than others for the development of mental illness before the presence of symptoms (Arango et al., 2018). Children of parents with mental illness face the highest cumulative risk of developing mental disorders (Uher et al., 2023). However, selective prevention for high-risk children yields variable outcomes across individuals (Lannes et al., 2021). This variability underscores the importance of applying precision prevention principles to determine the most effective interventions for high-risk groups.

Selective preventive interventions for families with parental mental illness aim to foster behavioral changes to mitigate modifiable risk factors and promote resilience in children. Examples include parenting skills training to improve parental behavior, psychoeducation to enhance communication, and training in adaptive coping skills (Marston et al., 2016). A systematic review of reviews has demonstrated that selective preventive interventions effectively reduce mental health problems in at-risk children and young people by promoting adaptive behaviors, enhancing coping mechanisms, and improving emotional regulation (McGovern et al., 2024).

However, despite their promising effectiveness, some children do not benefit from these interventions (Lannes et al., 2021). Selective prevention can reduce the risk of developing mental illness for children of parents with mental illness by about 50% (Lannes et al., 2021). This variation in efficacy may stem from the heterogeneity of the target group and their diverse and evolving needs (Lannes et al., 2021). Families with parental mental illness vulnerabilities and needs for support are diverse and complex (Duffy et al., 2023), which may lead to individualized intervention pathways and effect heterogeneity in selective preventive programs (Butler et al., 2020). Variations in intervention effectiveness stem from various areas. Differences in individual factors, e.g., perception of and experience with the intervention, as well as contextual factors, shape the response to an intervention (Michie et al., 2011). Understanding these variations requires investigating the underlying mechanisms that contribute to change.

Identifying mechanisms contributing to change can aid understanding of what works for whom and under which circumstances, to develop precision prevention and ultimately support children who do not benefit from the existing preventive interventions. These mechanisms are the contextual and individual components by which an intervention brings about change (Kazdin, 2007). The conceptualization of mechanisms varies across disciplines. In behavioral change science, they are often referred to as ‘mechanisms of action’ (MoAs) (Carey et al., 2018), whereas, in complex intervention research, they are often termed ‘mechanisms of change’ (Skivington et al., 2021). These definitions mostly encompass theoretical conceptualizations referring to quantitative (statistical) constructs, such as moderators and mediators that influence intervention outcomes (Kazdin, 2007). In qualitative research, mechanisms contributing to change are often defined as underlying contextual and individual processes contributing to outcomes when interacting with the intervention (Bonell et al., 2022).

In this study, we define mechanisms contributing to change as the contextual and individual mechanisms that interact with the intervention to facilitate change. We adopt the term to bridge both qualitative and quantitative research paradigms. Combining and integrating qualitative and quantitative approaches to understand underlying contextual processes can enhance identifying mechanisms contributing to change (Skivington et al., 2021).

Despite its potential, the literature on mechanisms contributing to change in selective preventive interventions for children of parents with mental illness is scarce (Lannes et al., 2021). In both quantitative and qualitative literature, evidence on potential moderators, mediators, and mechanisms of change remains inconclusive and understudied, possibly due to population heterogeneity, intervention complexity, and small sample sizes (Argent et al., 2020). Nevertheless, findings from a previous trial suggest that parental cognition, parenting behavior, and child coping may mediate changes for children participating in selective preventive interventions for children at familial risk for depression (Löchner et al., 2023). A qualitative study found that fostering parental agency in a parenting intervention for families affected by parental mental illness served as a mechanism for changing parenting behavior (Goodyear et al., 2022).

In this study, we aim to explore mechanisms contributing to change in the context of a family-based selective preventive intervention (the VIA Family intervention), with a particular focus on parents’ and children’s experiences. Specifically, we aimed to leverage qualitative data of perceived mechanisms contributing to change to identify moderators that could inform exploratory subgroup analyses.

## Methods

### Exploratory Sequential Mixed Methods Design

The current study is an exploratory sequential mixed methods study of three parts (Creswell & Plano Clark, 2018) (see supplemental material 1, available online). Part 1 is a qualitative study, based on abductive analysis of data generated from focus-group discussions. Part 2 builds on these findings to define a new variable for a quantitative analysis. Part 3 is a quantitative post hoc subgroup analysis based on secondary data from the VIA Family trial (Müller et al., 2019, 2024). We followed the GRAMMS guideline for the reporting of mixed methods studies (see supplemental material 2, available online) (O’cathain et al., 2008). The following section will describe the methods and results of the studies in their sequential order.

### Secondary Data from the VIA Family Trial

We used secondary data from the VIA Family trial (Müller et al., 2019, 2024). The trial compared the efficacy of a preventive intervention (VIA Family) with treatment as usual (TAU) (Müller et al., 2019). Participants were families with parental severe mental illness (recurrent major depression, bipolar disorder, or schizophrenia spectrum disorder). The intervention aims to decrease the impact of modifiable risk factors and increase protective factors for children with an elevated familial risk of developing mental illness (Müller et al., 2019). Each family received a case manager for 18 months, working within a multidisciplinary team highly experienced with mental health or social services. After introductory sessions with family-focused psychoeducation and identification of individual needs, the intervention was individually tailored based on the family’s needs and preferences, including sessions for parents, children, and the whole family. Key components were parenting support, social/legal support, peer support groups for children and parents, and early support for child mental health problems (Müller et al., 2019). Families in the TAU group accessed standard support available through social and mental health services (Gjøde et al., 2024; Müller et al., 2024). We tested the interventions in a randomized clinical trial in Denmark between 2017 and 2022. At baseline, 95 families with 113 children participated in the trial (Müller et al., 2024).

## Part I: Qualitative Study

### Part I Methods

#### Sampling and Data Generation

The qualitative sample included parents and children from the VIA Family group. Data generation took place in September 2021, during the COVID-19 pandemic. We facilitated four focus group discussions—two with parents (three to five per group) and two with children (four per group). Participants were selected from the intervention’s peer support groups, as these incorporated core elements of the intervention (e.g., psychoeducation, parenting support, and adaptive coping strategies). This helped us explore mechanisms contributing to change within the intervention despite the individually tailored design. To ensure comparable experiences, we invited families at the end of their intervention period. Using pre-existing peer support groups facilitated open discussions, as familiarity among participants enhances engagement and data richness in focus group research (Freeman, 2006). This was especially important for capturing children’s perspectives (O’Reilly & Parker, 2014). Due to pandemic-related social gathering restrictions, families from the TAU group and those in the VIA Family group without regular social contact were excluded. Two independent graduate students moderated the focus groups using a semi-structured theme guide and participatory methods (see supplemental material 3, available online). We audio-recorded the discussions and transcribed the complete sessions verbatim (O’Reilly & Parker, 2014).

Within the parents’ focus groups, five participants were mothers, and three had a severe mental illness diagnosis. The children’s focus groups included children aged 8–14 years, with five children identifying as female and three as male. We pseudonymized all names in the results section.

#### Qualitative Data Analysis and Theory

We conducted an abductive analysis following Timmerman and Tavory (Timmermans & Tavory, 2022). This iterative process integrates theory and empirical data to explore surprising findings (Timmermans & Tavory, 2022). Four researchers analyzed the data. We used consensus coding to ensure systematic coding. Coding disagreements were resolved through dialogue and reflection on clinical experiences and theory. The analysis commenced with individual open coding rounds of all transcripts. Researchers reviewed transcripts and audio recordings without a predefined theoretical framework. The focus at this stage was to develop codes that emerged directly from the empirical data, initially guided by the research question: *What mechanisms, if any, do participants experience as contributing to changes within the intervention?* The research team then discussed the initial codes to compare interpretations and identify emerging patterns of surprising findings.

After the initial coding, we introduced a theoretical framework to refine the analysis. To align with our definition of mechanisms, we applied two behavior change theories that conceptualize mechanisms of change as both contextual and individual processes: the health belief model (HBM) (Rosenstock, 1974) and the self-determination theory (SDT) (Ryan & Deci, 2000). According to HBM, individuals engage in health-preventive behaviors when they perceive a health threat as serious (severity) and likely to affect them (susceptibility) and when the perceived benefits of action outweigh potential barriers (Green et al., 2020; Rosenstock, 1974). Additionally, HBM highlights the role of ‘cues to action’—external or internal triggers that prompt behavior change. SDT emphasizes that people strive for basic psychological needs: autonomy, competency, and relatedness. These needs influence peoples’ motivation to engage and sustain in behavioral change (Ryan, 2023; Ryan & Deci, 2000). SDT categorizes motivation to behavioral change on a continuum from extrinsic to intrinsic, depending on the degree of autonomy involved. Supporting autonomy enhances motivation and engagement in behavioral change (Ryan & Deci, 2000). The social and cultural context shapes motivation by either supporting or thwarting the fulfillment of these basic psychological needs.

After integrating the theoretical perspectives into the analysis, we conducted a focused coding phase to systematically examine patterns and variations within the surprising themes from the initial coding using HBM and SDT (Tavory & Timmermans, 2014). In this phase we focused on coding within the surprising themes, analyzing patterns and variations using a modified research question: “How do the surprising themes impact and contribute to the experience of change?”.

#### Reflexivity

The final stage of the analysis involved a reflexive engagement between data, theory, and our background and expertise. As white female academics and mothers from high-income countries specializing in mental health and prevention, we both hoped for and believed in the value of preventive programs for at-risk children, and we were initially surprised by the preventive intervention’s limited effect on primary and secondary outcomes in the VIA Family trial(Gjøde et al., 2024; Müller et al., 2024). We continuously examined how our assumptions shaped the formation of the research questions, our analysis, and our interpretation.

## Part I Results: Qualitative Findings

**Fig. 1:**
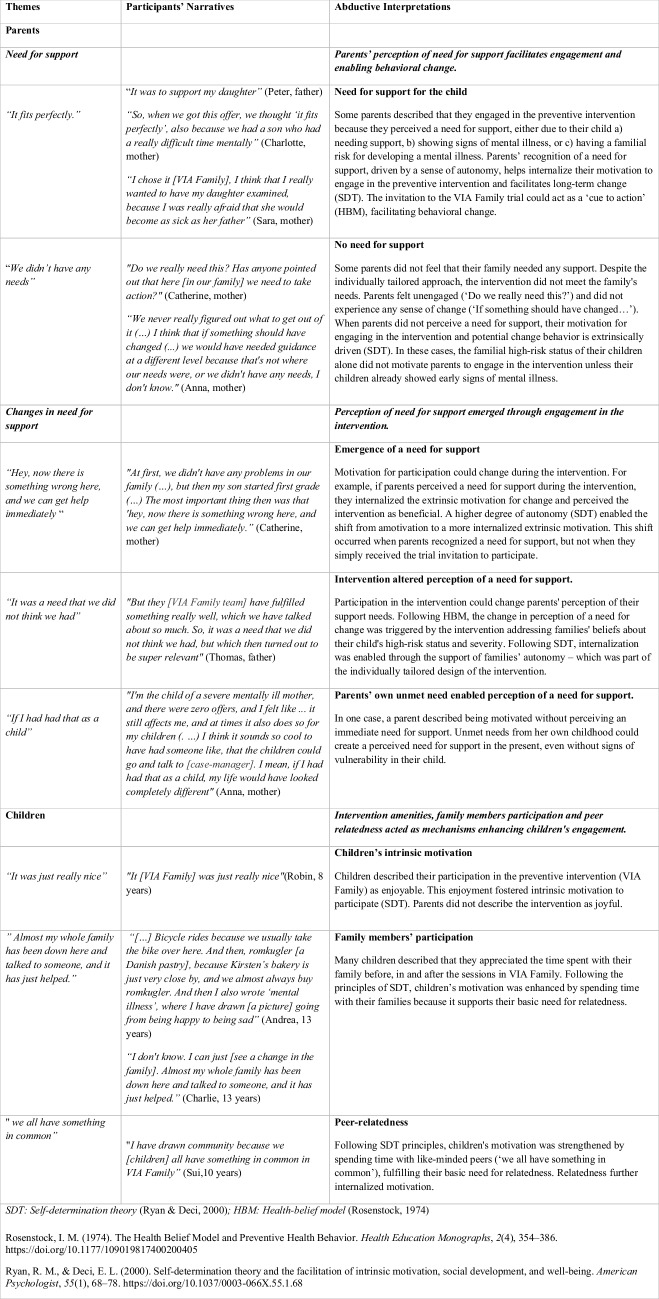
Summary of findings from the part I qualitative study

A summary of findings of the abductive analysis of parents and children’s focus group discussions is in Fig. 1.

### Parents: Perceived Need as Mechanism for Engaging in the Preventive Intervention

For the parents, perceiving a need for support served as a mechanism for engaging with the intervention. The ‘perception of need’ was enabled by (1) the child’s mental health problems, (2) the participation in the intervention, and (3) the parent’s own unmet need.

1. Perceived need enabled by child mental health problems: ‘It fits perfectly’ vs. ‘We didn’t have any needs’.

Perceiving a need for support: ‘It fits perfectly’.

Sara was the only parent who described that she initially engaged in the trial due to concerns about her daughter’s familial risk:

“I chose it [VIA Family trial], I think that I really wanted to have my daughter examined, because I was really afraid that she would become as sick as her father” (Sara, mother).

She describes here the target of selective preventive interventions: preventive support for populations at higher risk than others. However, for most parents it was not the ‘susceptibility’ (HBM) to mental health problems but the *presence* of mental health problems that prompted engagement in the intervention:

“So, when we got this offer, we thought ‘it fits perfectly’, also because we had a son who had a really difficult time mentally” (Charlotte, mother).

Charlotte engaged with the intervention because she perceived that her son had mental health problems, i.e. a serious health threat (severity) (HBM). The invitation to the trial (“when we got this offer”) then acted as a ‘cue to action’ (HBM). For Charlotte, autonomy (as the experience of volition and willingness of one’s actions) (SDT) was key to promoting her engagement with the intervention. She chose to participate because *she* perceived a need for support. The same was described by another parent, Catherine:

“At first, we didn’t have any problems in our family. […] but then my son started first grade […] The most important thing then was that ‘hey, now there is something wrong here, and we can get help immediately’.” (Catherine, mother).

Initially lacking internalized motivation, Catherine’s perspective changed when she personally recognized a need for support. Motivation for participation here becomes internalized. The change from amotivation to a more internalized extrinsic motivation is enabled by a higher degree of autonomy.

For both Catherine and Charlotte, perceiving a need for support for child mental health problems facilitated the internalization of motivation, a key factor in SDT that can foster sustainable change, aligning with the goals of preventive programs.

Absence of perceived need for support: ‘We didn’t have any needs’.

However, not all parents observed early signs of mental health problems or vulnerability in their children. This absence often hindered engagement and the possibility of experiencing change:

“We never really figured out what to get out of it […] I think that if something should have changed […] we would have needed guidance at a different level because that’s not where our needs were, or we didn’t have any needs, I don’t know.” (Anna, mother).

Anna’s lack of perceived need led to her disengagement with the intervention; she “never really figured out what to get out of it”. This contrasts with the aim of selective prevention. Selective preventive interventions aim to target individuals at high risk. However, in Anna’s case, her child simply having a high-risk status (susceptibility) was not enough to motivate engagement and facilitate change.

(2) Perceived need enabled by the intervention: ‘It was a need that we did not think we had’.

The intervention itself could also facilitate internalization and thereby engagement, as noted by Thomas:

“But they [VIA Family team] have fulfilled something really well, which we have talked about so much. So, it was a need that we did not think we had, but which then turned out to be super relevant” (Thomas, father).

Many parents described that their motivation became internalized through the intervention, as they recognized a new or previously unacknowledged need. This change in motivation can be explained by the intervention addressing the child’s high-risk status (susceptibility) and severity—key concepts from HBM explaining why people engage in preventive behavior. Additionally, according to SDT, this shift in engagement is facilitated by autonomy, i.e., when parents perceive that they have a need. This highlights how selective preventive interventions can promote engagement by addressing parents’ perception of the need for support, thereby shifting motivation from extrinsic to intrinsic.

(3) Perceived need enabled by parents’ own unmet need: ‘If I had had that as a child’.

In all but one of the above examples, parents felt the intervention was relevant and could contribute to change when there was an actualized perceived need. In these cases, the selective preventive intervention was used as an early intervention for a perceived need, rather than its intended purpose of preventing problems before they arise.

However, one parent described that motivation to engage stemmed from her own unmet needs from childhood, rather than from identifying a need for support for her child. This unmet need prompted the perception of a mirroring perceived need for her child.

“I’m the child of a severely mentally ill mother, and there were zero offers, and I felt like… it still affects me, and at times it also does so for my children […] I think it sounds so cool to have had someone like, that the children could go and talk to [case-manager]. I mean, if I had had that as a child, my life would have looked completely different.” (Anna, mother).

Anna’s motivation was influenced by her personal history of growing up with parental mental illness. This history drives her autonomy and the motivation to provide her children with the support she lacked.

### Children: The Role of Relatedness and Intrinsic Motivation in Engagement

Contrary to parents, children did not discuss perceived needs as reasons for engagement. Instead, they were driven by intrinsic motivation (SDT), often linked to enjoyable activities, becoming part of a group with children with similar experiences, and family involvement:

“It [VIA Family intervention] was just really nice”(Robin, 8 years).

Most children described how contextual factors of the intervention, especially family-related activities in context with the intervention, were significant motivators. When asked to describe and draw what VIA Family meant for them, Andrea, for example, said:

“Bicycle rides, because we usually take the bike over here. And then, romkugler [a Danish pastry], because Kirsten’s bakery is just very close by, and we almost always buy romkugler. And then I also wrote ‘mental illness’, where I have drawn [a picture of] moving from being happy to being sad” (Andrea, 13 years).

Andrea describes how the preventive intervention enabled the family to engage in joint activities: riding the bike and buying pastries. Andrea associated these family activities as much with the intervention as learning about mental illness. Several children echoed the importance of the time spent with their family before, during, and after the intervention. Being together with the family fostered relatedness, which is a core concept in SDT to drive internalized motivation. When asked about changes in the family after enrolling in the intervention, Charlie responds:

“I don’t know. I just can [see a change in the family]. Almost my whole family has been down here and talked to someone, and it has just helped.” (Charlie, 13 years).

Relatedness was fostered through family engagement but also through meeting other children. When asked to describe the intervention, Sui said:

“I have drawn community because we [children] all have something in common in VIA Family. We talk about all the things that are on our minds and that we want to discuss […] there is a reason we are here, and that is what we all have in common” (Sui,10 years).

Following the principles of SDT, we can understand the children’s motivation as enhanced by spending time with their families and like-minded peers (“we all have something in common”) because they support their basic need for relatedness. Through participation in the intervention, the children’s motivation moved from extrinsic (motivated by their parent’s decision to participate) to intrinsic (motivated by enjoyable activities and relatedness).

## Summary of Qualitative Findings

For parents, perceiving a need for support was crucial for engaging with the intervention. While one parent linked this need to their child’s familial high-risk status, most were driven by (a) their child’s current mental health problems, (b) participation in the intervention, or (c) an unmet need from their past. For children, intrinsic motivation was fostered through (a) the intervention cultivating relatedness with peers and family, and (b) engagement in familial activities before, during, and after sessions. For children, intrinsic motivation was fostered through (a) the intervention cultivating relatedness with peers and their families, and (b) the activities they engaged in before, during, and after intervention sessions. In both cases, as motivation became more internalized (SDT)—whether through recognizing the need for support (parents) or social activities and relations (children)—participants engaged more deeply. These mechanisms of internalization and engagement are key contributors to change, aligning with SDT’s framework of autonomous motivation for lasting impact.

## Part II: Mixed Methods Integration

### Methods and Results Part II

In Part II, we integrated findings from Part I with the objectives of Part III. Using a sequential design, we moved from qualitative findings (participants’descriptions) to quantitative testing (see supplemental material 1, available online). Parents’ narratives helped identify a variable for subgroup analyses in Part III, potentially serving as a mechanism contributing to change in the preventive intervention VIA Family. This approach systematically transitioned from understanding participants’ experiences to measuring their quantitative impact.

Part I revealed that the type of motivation to participate influences parental engagement with the intervention. We translated this finding into a’motivation for participation’ variable using secondary data from the VIA Family trial. In the original trial, a qualitative variable on parents’ reason for participation was included to inform the intervention team if families were randomized to the experimental group. The baseline assessment team categorized participants’ responses during the interview as, e.g., wanting to ‘support science’ or specific support areas (e.g., parenting advice, child mental health problems) (see supplemental material 4, available online). If families cited multiple reasons, they identified the most prominent one. For the current study, we categorized parents’ reasons for participating into two groups: those motivated by a desire to contribute to science and those seeking support for a need (see supplemental material 4, available online).

## Part III: Quantitative Study

### Part III: Methods

#### Sampling and Data Collection

We analyzed a subsample of participants from the VIA Family trial using secondary data (Müller et al., 2019). Families were eligible for the subsample if they reported their reasons for participating in the trial at baseline. We included the trials’ primary and secondary outcome measures (Müller et al., 2019): changes on the Children’s Global Functioning Scale (CGAS) (Shaffer, 1983) and the Home Observation for Measurement of the Environment (HOME) Inventory (Caldwell & Bradley, 2003). Whereas the CGAS assesses the lowest level of a child’s psychosocial functioning over the past 30 days across home, school, and leisure settings based on clinical interviews with the parent and child, the HOME Inventory evaluates the quality of the child’s home environment through a combination of interviews and direct observation, focusing on cognitive stimulation, emotional support, parental responsiveness, and the presence of developmentally appropriate materials and experiences (Caldwell & Bradley, 2003; Shaffer, 1983). Higher scores indicate higher global functioning on the CGAS and a more supportive home environment on the HOME Inventory. CGAS inter-rater reliability is moderate and HOME inter-rater reliability is acceptable (Glad et al., 2014; Lundh et al., 2016).

#### Statistical Analyses

We conducted post hoc subgroup analyses to explore whether the effect of the intervention varied according to reasons for participation (see Part II). To ensure consistency and comparability, we repeated the analyses of the primary outcome (CGAS) and the secondary outcome (HOME) in each subgroup in turn using the same model as was used in the original trial (Müller et al., 2019). This was a linear mixed model with the change in CGAS/HOME from baseline to post-intervention as the outcome and including allocation group, gender, age, and TOMAL immediate scores as fixed effects together with a random effect of family to account for correlation between outcomes from siblings. All analyses were performed in accordance with the intention-to-treat (ITT) principle, including all participants from the relevant subgroup regardless of treatment adherence and study completion. Missing data were handled by 100-fold multiple imputations, which were run for 20 iterations to ensure convergence. Results are presented in forest plots showing subgroup-specific adjusted mean treatment differences with 95% confidence intervals. Owing to the post hoc exploratory nature of the analyses, no adjustment for multiple testing was applied. Hence, results should be interpreted with caution as false positives cannot be ruled out. We conducted a sensitivity analysis to account for the potential confounding effect of baseline score differences between subgroups. All analyses were conducted using R statistical software version 4.2.2, utilizing the packages ‘lme4’ (Bates et al., 2015), ‘mice’ (Buuren & Groothuis-Oudshoorn, 2011), and ‘Amelia’ (Honaker et al., 2011).

### Part III: Results

We included data from 110 children and their primary caregivers in the analyses. We excluded three children from the trial in the analysis, with no parental data on reasons for participation. The subgroups showed that 40 (43%) primary caregivers (of 48 [43.6%] children) were primarily motivated to participate in the study because they wanted to help science, and 53 (57.0%) primary caregivers (of 62 [56.4%] children) wanted support. Within the TAU group, there are different baseline scores on the HOME and CBCL. These differences are not observed in the VIA Family subgroups. See Table 1 for demographic and clinical characteristics of the participating children and their parents in the subgroups for group VIA Family and TAU.

**Table 1:** Baseline demographic characteristics divided by intervention group and primary caregiver’s main reason for study participation

	VIA Family		Treatment as usual	
Main reason for participation	Contributing to science	Wanting support	Contributing to science	Wanting support
*All families*	*N* = 46		*N* = 47	
Primary caregiver, *n* (%)	20 (43)	26 (57)	20 (43)	27 (57)
Having a SMI diagnosis^a^	15 (33)	18 (39)	15 (32)	20 (43)
Personal and Social Functioning (PSP) (Michalos, 2014)	68.30 (11.88)	66.96 (15.75)	65.30 (17.46)	70.26 (15.28)
*All children*	*N* = 56		*N* = 54	
Children, *n* (%)	24 (43)	32 (57)	24 (44)	30 (56)
Children’s age at baseline in years, mean (SD)	9.5 (2.1)	9.9 (1.7)	9.5 (2.2)	9.5 (1.8)
Biological sex female, *n* (%)	10 (42)	14 (44)	11(46)	17 (53)
Global functioning (CGAS) ( Shaffer, 1983 ), mean (SD)	64.79 (13.11)	67.09 (13.21)	62.38 (15.47)	65.00 (15.07)
Home environment (HOME_)_( Caldwell & Bradley, 2003 ), mean (SD)	47.44 (4.44)	46.26 (6.62)	44.67 (6.70)	48.61 (4.48)
Behavioral or emotional problems (CBCL) ( Achenbach, 2001 ), T-score, mean (SD)	56.54 (10.44)	55.86 (13.20)	68.17 (18.39)	56.88 (14.89)

^a^*SMI* Severe Mental Illness

Achenbach, T. M. (2001). Manual for ASEBA school-age forms & profiles. *University of Vermont, Research Center for Children, Youth & Families; *Caldwell, B. M., & Bradley, R. H. (2003). Home observation for measurement of the environment: Administration manual. *Tempe, AZ: Family & Human Dynamics Research Institute, Arizona State University*; Michalos, A. C. (Ed.). (2014). PSP Scale. *Encyclopedia of Quality of Life and Well-Being Research* (pp. 5134–5134). Dordrecht: Springer Netherlands; Shaffer, D. (1983). A Children’s Global Assessment Scale (CGAS). *Archives of General Psychiatry, 40*(11), 1228

**Fig. 2:**
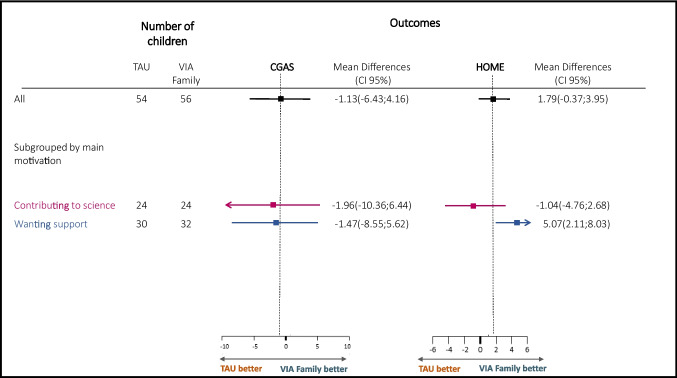
Comparison between differences in mean change at post-intervention for treatment as usual and VIA Family on CGAS and HOME, subgrouped by main motivation for study participation at baseline. Higher scores indicate higher global functioning (CGAS) and higher quality of a supportive home environment (HOME)

Subgroup analyses of families wanting support at baseline showed no significant differences between groups at post-intervention in children’s global functioning measured with CGAS (− 1.47, 95% CI − 8.55 to 5.62). However, regarding a change in levels of support and stimulation in the home environment, there was an effect for the VIA Family with a clinically meaningful difference for families seeking support at baseline (5.07, 95% CI 2.11 to 8.03) (Fig. 2). Results remained unchanged after controlling for baseline differences between subgroups as potential confounders.

## Discussion

This study aimed to explore mechanisms contributing to change in a selective preventive mental health intervention for high-risk children using qualitative and quantitative data in a sequential exploratory mixed methods design. We used focus groups and secondary data from the VIA Family trial (Müller et al., 2019, 2024). Findings from the qualitative analysis revealed that parents’ perceived need for support and children’s strive for relatedness facilitated engagement in the intervention. The parental findings informed a statistical exploratory subgroup analysis that showed that families from the intervention group who joined because they perceived a need for support significantly improved their home environment (quantitative study) compared with those motivated by contributing to science, but the analysis showed no significant differences in children’s global functioning measured with CGAS.

This variation in outcomes suggests that the effectiveness of a preventive intervention depends not only on its content but also on contextual factors such as perceived need for support and relatedness. Specifically, parents with a perceived need for support showed greater engagement (qualitative findings) and improvements in the home environment (quantitative results). This aligns with previous studies showing that the presence of child mental health problems is strongly associated with parental enrollment in preventive mental health interventions (Houle et al., 2022) and parenting interventions (Finan et al., 2018). Parents who actively seek support may be more receptive to guidance and more likely to adopt intervention content, e.g., positive parenting strategies. Moreover, changes in the home environment are more directly influenced by parents themselves, e.g., establishing a fairly regular and predictable daily schedule for the child (Item 1 in the HOME inventory; Caldwell & Bradley, 2003), whereas improvements in children’s global functioning (measured with CGAS) also depend on broader contexts, e.g., the school context, that the intervention does not directly target. This could help explain why we observed significant changes in HOME, but not in CGAS scores. Nevertheless, improvements in the home environment could precede changes in child functioning (Van Der Put et al., 2018), and we are currently exploring the long-term effects of the intervention in a follow-up study.

Beyond this, the qualitative findings revealed several additional mechanisms contributing to change that we could not test using the available quantitative data. Contrary to their parents, children described peer and family relatedness as mechanisms contributing to change. These findings align with research linking peer relationships to mental well-being and self-esteem (Pollak et al., 2023). The findings from children’s focus groups also suggest that positive family experiences, such as joint activities, may be key drivers of change, consistent with the research literature on the positive role of family dynamics in prevention programs (Goodyear et al., 2022; Guzman Holst et al., 2023).

Our findings on perceived need, motivation, and family and peer relatedness highlight the need to further explore these mechanisms using qualitative and quantitative methods. Especially, child-driven mechanisms, such as social relatedness, warrant further investigation to determine their contribution to long-term intervention effects. Expanding the scope of future studies to include these mechanisms would provide a richer understanding of how selective prevention interventions can address the diverse needs of high-risk populations. By identifying mechanisms contributing to change, our findings contribute to the field of precision prevention, informing the design of tailored preventive interventions that align with individual family needs and characteristics.

This study has some limitations. First, the qualitative sample lacked ethnic diversity, despite its presence in the VIA Family trial and our efforts for inclusion, which may limit the generalizability of our findings. Nevertheless, our qualitative sample was diverse in terms of other important social determinants of mental health such as socioeconomic background, history of mental illness, age, and gender (Kirkbride et al., 2024). Second, we interviewed only families still engaged in the intervention, which may have emphasized immediate experiences over long-term perspectives. The identified mechanisms may reflect those occurring during the intervention but not capture mechanisms contributing to sustained change. Moreover, COVID-19 restrictions further limited participation in intervention families with peer support group experiences, excluding TAU families. While this reduced comparative perspectives, it ensured compliance with public health guidelines and focused on exploring mechanisms within the intervention. These factors may limit generalizability and introduce potential biases. Third, although children actively participated in the focus group discussions, their ability to articulate their experiences may have been constrained by age-related verbal communication skills. Moreover, translating quotes from native Danish to English dismisses a cultural-specific understanding of language and meanings. Fourth, our theoretical framework may not fully capture the complexity of engagement and behavior change, as it is influenced by many social and cultural factors (Reupert et al., 2021). Moreover, participants may have overreported their motivation as ‘contributing to science,’ a socially desirable response compared to less favorable reasons, such as ‘needing support with family problems.’ Nevertheless, previous research has shown that contributing to science and altruism are important facilitators for study participation (Sheridan et al., 2020). Lastly, the post hoc subgroup analyses were exploratory, and results should be interpreted with caution. The small sample size limits statistical power, increasing the risk of false positives and negatives, and we cannot exclude a confounding effect of differential dropout. Consequently, these exploratory findings should be used to generate hypotheses for future studies with larger samples, which could confirm subgroup effects and better account for differential dropout.

Despite its limitations, our study has several strengths. The sequential mixed methods design enabled us to translate qualitative findings into new hypotheses for quantitative testing, strengthening theory development. Moreover, the qualitative findings nuance previous findings by exploring factors that increase engagement in families where children do not exhibit mental health problems. By including both parents’ and children’s perspectives, we were able to compare their experiences, offering insight into how family engagement mechanisms interact.

Future studies could explore the role of motivation and relatedness in selective prevention for mental health. Despite its popularity in intervention research, the role of motivation has received limited attention in selective preventive mental health interventions (Pas & Bradshaw, 2021). These interventions target populations that are at high risk but do not show symptoms yet. However, our findings suggest that the absence of child mental health problems in selective prevention may hinder effective engagement in those programs. Moreover, children’s high-risk status alone (without the presence of symptoms) may only serve as an extrinsic motivation for engagement, which may lead to low parental participation in preventive programs (Houle et al., 2022). Ultimately, a lack of intrinsic motivation could hinder both initial enrollment and sustained engagement, reducing the likelihood of lasting behavioral change. A stronger focus on intrinsic motivation in selective prevention programs may enhance engagement, particularly in families where children exhibit only subtle or no symptoms, or in the earliest stages—pregnancy, infancy, and toddlerhood—when symptoms are not yet visible but targeted interventions may have the greatest long-term impact. Furthermore, future research could explore ways to enhance children’s motivation and engagement in selective preventive interventions. Investigating child-specific mechanisms could contribute to the design of more effective selective prevention. In particular, social relatedness is recognized as an important protective factor for children of parents with a mental illness (Piché et al., 2024; Van Schoors et al., 2023). Future studies could examine how children’s sense of relatedness functions as a potential moderator or mediator of treatment effectiveness. Finally, while our study does not explicitly test the identified mechanisms as mechanisms of action (MoAs), our findings align with recognized MoAs associated with behavioral change, such as motivation and social influences (Carey et al., 2018). Future research could examine their role as mediators of intervention effects within selective interventions with structured behavior change models.

Our findings have several implications for practice. First, addressing parents and children’s intrinsic motivation could improve engagement in selective preventive interventions and ultimately enhance their efficacy. One way is to incorporate motivation theories to guide enrollment strategies (Finan et al., 2018). Another way is by integrating aspects of relatedness (e.g., family activities and peer support) to enhance children’s engagement (Ryan & Deci, 2000). Moreover, regular assessments, evaluations, and discussions with patients in the mental health services about how their mental illness affects their next-of-kin and potential needs for support in the family could motivate parents to engage in selective preventive strategies (Festen et al., 2014). Additionally, a stepped-care model tailored to motivation levels and perceived need for support could improve engagement in preventive interventions for this population. In this model, autonomy-respecting and least intrusive programs, such as bibliotherapy and (self-help) online interventions for children at risk of mental illness, could serve as the first step for some families with parental mental illness, helping them engage and recognize needs they had not identified (Villatte et al., 2023). These suggestions are supported by the findings of a recent review concluding that online programs could increase the engagement of non-engaged parents in preventive interventions (Finan et al., 2018). Lastly, not all children at familial risk for mental illness will develop a mental illness (Uher et al., 2023). Research in high-risk populations is essential to target the right at-risk group and motivate them to engage in preventive interventions.

## Supplementary Information

Below is the link to the electronic supplementary material.

ESM 1 (PPTX 39.9 KB)

ESM 2 (DOCX 14.9 KB)

ESM 3 (DOCX 27.9 KB)

ESM 4 (DOCX 19.2 KB)

## Data Availability

Data are partly available upon reasonable request.
