# Supplementary material for: Perceived Need and Social Relatedness Contribute to Change in Selective Prevention for Mental Illness: a Mixed Methods Study
Source: Prev Sci. 2025 Aug 12;26(6):908–20. doi: 10.1007/s11121-025-01831-w (PMC12394378; doi:10.1007/s11121-025-01831-w)
Supplement: Supplementary file 1 — (PPTX 39.9 KB) [file 11121_2025_1831_MOESM1_ESM.pptx]

## Slide 1
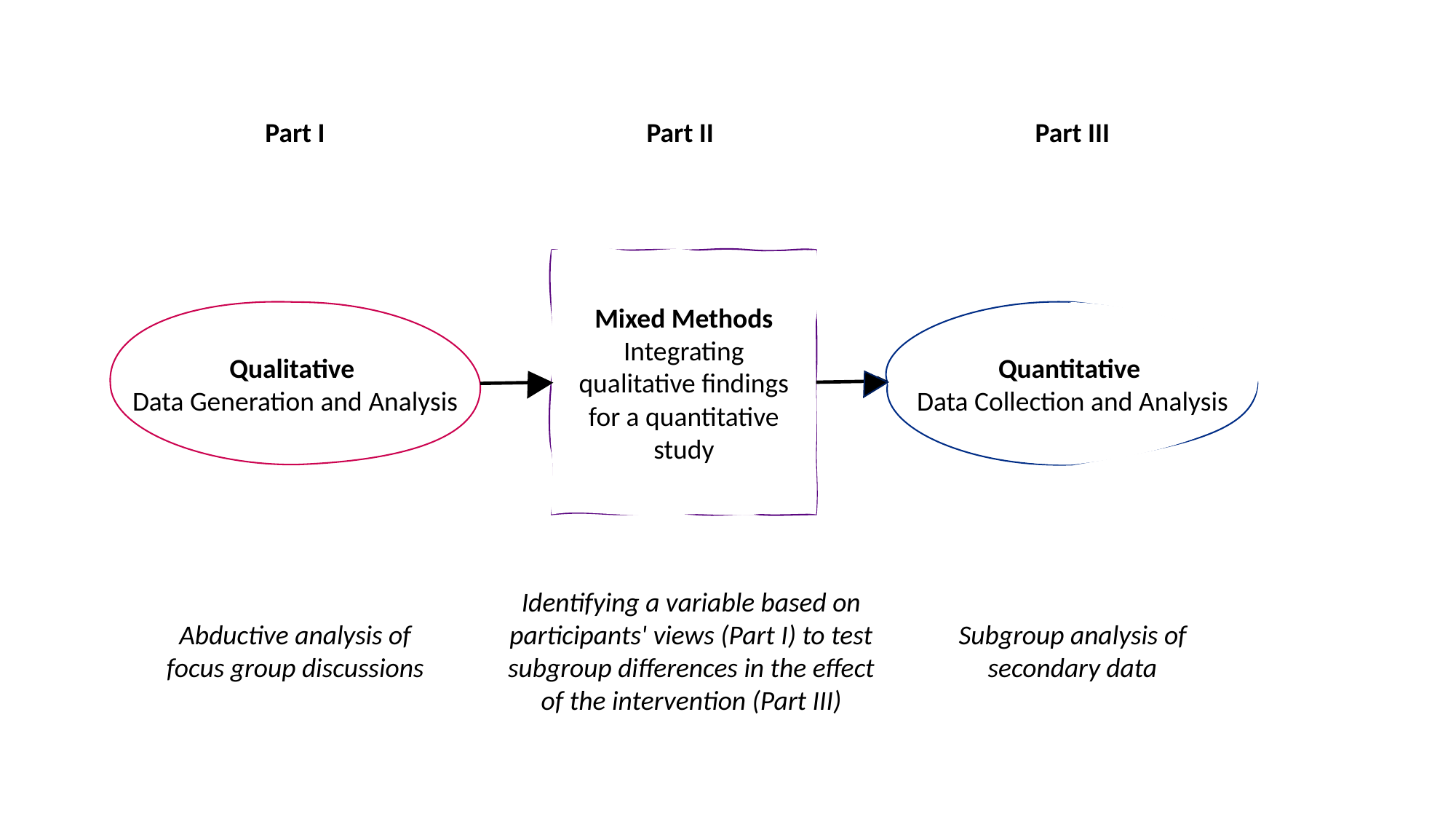

Part I
Part II
Part III
Mixed Methods Integrating qualitative findings for a quantitative study
Qualitative
Data Generation and Analysis
Quantitative
Data Collection and Analysis
Identifying a variable based on participants' views (Part I) to test subgroup differences in the effect of the intervention (Part III)
Abductive analysis of focus group discussions
Subgroup analysis of secondary data
