# Supplementary material for: Perceived Need and Social Relatedness Contribute to Change in Selective Prevention for Mental Illness: a Mixed Methods Study
Source: Prev Sci. 2025 Aug 12;26(6):908–20. doi: 10.1007/s11121-025-01831-w (PMC12394378; doi:10.1007/s11121-025-01831-w)
Supplement: Supplementary file 2 — (DOCX 14.9 KB) [file 11121_2025_1831_MOESM2_ESM.docx]

| Guideline | Section and Page |
| --- | --- |
|  |  |
| 1) Describe the justification for using a mixed methods approach to the research question | Introduction, page 4. Methods, page 5. |
| 2) Describe the design in terms of the purpose, priority and sequence of methods | Methods, page 5. |
| 3) Describe each method in terms of sampling, data collection and analysis | Methods, page 5-7 and 11. |
| 4) Describe where integration has occurred, how it has occurred and who has participated in it | Methods, page 11, Discussion, page 14 and 17 (conclusion) |
| 5)Describe any limitation of one method associated with the present of the other method | Discussion, page 16 and 17. |
| 6) Describe any insights gained from mixing or integrating methods | Discussion, page 14 and 17 (conclusion) |

Supplement 1: GRAMMS guidelines

O'Cathain A, Murphy E, Nicholl J. The quality of mixed methods studies in health services research. J Health Serv Res Policy. 2008;13: 92-98
