# Supplementary material for: Perceived Need and Social Relatedness Contribute to Change in Selective Prevention for Mental Illness: a Mixed Methods Study
Source: Prev Sci. 2025 Aug 12;26(6):908–20. doi: 10.1007/s11121-025-01831-w (PMC12394378; doi:10.1007/s11121-025-01831-w)
Supplement: Supplementary file 3 — (DOCX 27.9 KB) [file 11121_2025_1831_MOESM3_ESM.docx]

Supplement 2: Guide Focus Group Discussion (translated from Danish by ADM)

***Parents***

**Agenda (30 min)**

Welcome: Information and Consent Forms

Exercise 1: Introduction round with picture cards

Exercise 2: My Journey through VIA Family

*Break*

Exercise 3: Draw-a-Question

Thank you and Goodbye

**Welcome:** Information and Consent Forms (10:00-10:10)

Welcome, and thank you for joining us today. I am K, and along with S, I am involved with the VIA Family study as part of our anthropology thesis.

We have invited you here today because we want to hear about your experiences with being part of VIA Family. The information you share today will be used in the VIA Family Evaluation Study. To begin, I would like to pass around some consent forms. We hope you will sign them before we proceed. With these consent forms, you permit us to use the information you provided today in the evaluation. Any information you share will, of course, be anonymised. *Start the recorder and go through the agenda.*

Before we start, I want to emphasise that it's entirely up to you what and how much you want to share today. If there is something you do want to talk about, that is totally ok. I would also like to mention that the purpose of today's focus group is not necessarily to paint an overly positive picture of the VIA Family. We want to hear how you have experienced being a part of it, what has worked well, and what has not worked so well.

**Exercise 1:** Introduction round with picture cards (10 min)

As you can see, I have placed various picture cards on the table. What I would like to start with is for each of you to look at the different cards and choose one that best describes what VIA Family has been for you and your family. Then we will go around, and you can start by stating your name and explaining why you chose the card you did.

**Exercise 2:** My Journey through VIA Family (35 minutes)

With the next exercise, I want to gain insight into your journey through VIA Family. In this exercise, I will ask you to reflect on your entire experience with VIA Family, from the beginning when you were recruited for the project to now, where you (almost) completed your participation. I have created seven circles on the paper, and in each circle, you should note the answer to the corresponding question as indicated on the second piece of paper I have provided. This means that in circle number 1, you write the answer to question number 1. You will have 15 minutes for the exercise, after which we will discuss what you have noted. *The provided sheet*

*Before:* What were your initial thoughts when contacted by VIA Family?

Why did you choose to participate?

*During:* What has been helpful for you and your family?

What has not been helpful for you and your family?

What has been particularly challenging or difficult?

*After:* How do you feel about your participation in VIA Family coming to an end?

What is the most important thing you and your family will take away from this experience?

**BREAK: 10 minutes**

**Exercise 3**: Draw-a-Question (20 minutes)

I have now placed a bowl in the middle of the table containing slips of paper with questions. The idea is for each of you to take turns drawing a question. You will begin by answering the question you draw; others can contribute with their reflections.

***The Questions:***

• What has made the most impression on you during the process?

• What does it mean for you to have a contact person?

• If you were to tell a friend or colleague what VIA Family is, what would you say?

• How have you experienced your meetings with the researchers and the activities you have had to do when you have been at Frederiksberg Hospital?

• Has VIA Family been able to meet the needs that you and your family have had?

• Has it changed anything in your everyday life as a family to have been a part of VIA Family?

**Thank you and Goodbye** (5 minutes)

• Gift cards.

• You are always welcome to contact us if you have any questions.

***Children***

**Introduction:** What are we doing today? (5 minutes)

Presentation of facilitator.

“Today, let us pretend we are detectives on a mission. I want to know what it was like for you as a kid in VIA Family, and since you have experienced it, I think of you as an experts. So, can you bring me onto your team and tell me more about what VIA Family is and how it has been for each of you? Are you up for it?

When we are on a mission, there are usually tasks to do. Today, we have three tasks to complete. Let me tell you the plan. I want to hear your own stories and experiences. All experiences are important, whether they are good or a bit tough. You only share what you want to. I am recording to remember all the good things you tell me.”

**Agenda**

Welcome and Introduction Round

Task 1: Puzzle

Task 2: Plus and Minus

Break (10 minutes)

Task 3: Rope Activity

Thank you, and goodbye 😊

**Check-in-exercise:** picture cards (10 minutes)

| **Goal** | **Material** | **How-to** |
| --- | --- | --- |
| Creating a good and safe atmosphere among the children  This helps children open up in front of their peers in the focus group discussion. | Picture Card | The pictures are laid out on a table.  Each child chooses one card that depicts their experience in VF (likely referring to a specific context or place).  The children share their stories (cards). |

**Task 1:** Puzzle (20 minutes)

Introduction: When on a mission, people sometimes talk about certain pieces needing to be placed in a larger puzzle. The first task is about jointly putting together a puzzle. However, the pieces are blank, so we must draw something on them.

**Question:** *When I say VIA Family, what comes to your mind?*

| Goal | Material | How-to |
| --- | --- | --- |
| To gain an understanding of how the children perceive the VIA Family. | Puzzle pieces  Markers  Glue | The children each receive two puzzle pieces.  They draw what they associate with VF (presumably a specific context or place).  During the activity, the child shares their pieces, applies glue, and attaches them to a wall or board.  The task is considered completed when the puzzle has assembled all its pieces. |

Assistant questions:

• When you were in VF, what did you do?

• If you had to explain VF to a friend, what would you say?

• Is it something you have participated in as a family or something you have attended individually?

• Who are the people involved in VF?

• How has it been that it has occurred here in the house?

***Notes/observations:***

**Task 2: + and - (15 minutes)**

Introduction: I would really like to understand what you think has been good and what has not been so good about being involved in VF. That is what the next task is about.

| **Goal** | **Material** | **How-to** |
| --- | --- | --- |
| To gain insight into the children’s experience of being involved in VF. | Post-it  Markers | The children receive three Post-its:  “I think the best thing about VF has been that...”  “I haven’t liked that...”  “When I had to go to VF, I’ve had a feeling of...”  They write down everything they can think of, both positive and negative aspects of VF (5 minutes). Post-its are placed on the wall.  Now, let’s take a turn where I would like everyone to share what they have written down. |

Assisting questions:

Under ‘The best...’: Is it something you think you’ll miss?

Have you talked to your friends about what you’ve done in VF?

Have you discussed at home what you’ve done in VF?

***Notes/observations:***

**Task 3:** The Rope (20 minutes)

Introduction: We are soon through with our mission, but there’s one last task we need to complete. It can be challenging, so it is entirely okay if you need time to think. We need to go onto the floor.

You can stand where you feel it is right for you. It is completely okay to stand somewhere where no one else is standing**.**

| **Goal** | **Material** | **How-to** |
| --- | --- | --- |
| To gain an understanding of how the children have experienced VF affecting their family’s everyday life.  To show diversity.  To demonstrate development. | A rope/cord.  Papers with statements.  Printed paper with “Fits me very well” and “Doesn’t fit me at all.” | The rope is laid out on the floor.  The children line up and must position themselves on the rope according to how much they agree or disagree with the statements read aloud.  After the children have placed themselves on the rope, this is followed by a dialogue about why they chose to stand where they did. |

Statements:

• Sometimes, I think about what I have done or talked about in VF at home or school.

• I can feel a difference in how we are as a family after we have been in VF.

• I talk with my parents about different things than I did before we became part of VF.

• I talk with my friends about different things than I did before we became part of VF.

• I think something in my family has gotten worse by being a part of VF.

Assisting questions:

• Has VF changed anything in your and your family’s everyday life (at home, at school)?

• Is there anything you and your family have discussed/done in VF that you have used at home or school?

***Notes/observations:***

**Conclusion (11:20-11:30)**

The mission was successful! Thank you for your help and all the knowledge you shared today. You have been so awesome, and I have learned a lot. Here is a brief summary of what we have discussed.

Before we conclude, I have one last activity. Here is a wish box. You will each receive a small piece of paper with the question, “Would you wish for VF to continue?” You can write ‘yes’ or ‘no’ on the paper, and if you’d like, you can also write why you answered yes or no. Afterwards, you place your wish in the wish box. Once you’ve done that, we are finished for today.

Thank you and Goodbye
