# Supplementary material for: Perceived Need and Social Relatedness Contribute to Change in Selective Prevention for Mental Illness: a Mixed Methods Study
Source: Prev Sci. 2025 Aug 12;26(6):908–20. doi: 10.1007/s11121-025-01831-w (PMC12394378; doi:10.1007/s11121-025-01831-w)
Supplement: Supplementary file 4 — (DOCX 19.2 KB) [file 11121_2025_1831_MOESM4_ESM.docx]

| Original variable from baseline sociodemographic interview | Dichotomization of the continuous variable | New variable for subgroup analysis |
| --- | --- | --- |
| 1) Help Science |  | **Contributing to science** |
|  |  |  |
| 2) Get support for how we, as a family, are together |  | **Wanting Support** |
| 3) Get help with parenting/child rearing |  |  |
| 4) Concerned about child and wanting support or guidance for the child's well-being or mental health |  |  |
| 5) Get help on how to talk about mental illness in the family |  |  |
| 6) Other* |  | * |
| *If possible, category 6 was later categorized by the research team as either *contributing to science* or *wanting support*. If it was impossible to categorize, it was left as 'other' and not included. | | |
|  | | |

**Supplement 3**

*Figure S3 Identification and dichotomization of a variable for hypothesis testing in Part III*
